# Selection of the Better Dual-Timed Up and Go Cognitive Task to Be Used in Patients With Stroke Characterized by Subtraction Operation Difficulties

**DOI:** 10.3389/fneur.2020.00262

**Published:** 2020-04-23

**Authors:** Ampha Pumpho, Nithinun Chaikeeree, Vitoon Saengsirisuwan, Rumpa Boonsinsukh

**Affiliations:** ^1^Faculty of Physical Therapy, Srinakharinwirot University, Nakhonnayok, Thailand; ^2^Department of Physical Therapy, School of Integrative Medicine, Mae Fah Luang University, Chiang Rai, Thailand; ^3^Department of Physiology, Faculty of Science, Mahidol University, Bangkok, Thailand

**Keywords:** Timed Up and Go, cognitive, stroke, subtraction, phonologic fluency

## Abstract

**Background:** The Timed Up and Go Test (TUG) with serial subtraction is commonly used to assess cognitive-dual task performance during walking for fall prediction. Some stroke patients cannot perform number subtraction and it is unclear which cognitive task can be used to substitute for the subtraction task in the TUG test. The aim of this study was to determine the type of cognitive task that produced the highest decrease on both motor and cognitive performances during TUG-dual in stroke patients.

**Methods:** A total of 23 persons with stroke but capable of completing subtraction (ST) and 19 persons with subtraction operation difficulties (SOD) participated. Both groups have a similar age range (ST: 59.3 ± 10.4 years and SOD: 62.0 ± 6.8 years) and stroke onset duration (ST: 44.13 ± 62.29 months and SOD: 42.34 ± 39.69 months). The participants performed TUG without a cognitive task (TUG-single) followed by a cognitive task when seated (cognitive-single). In addition, TUG with a cognitive task (TUG-dual) was performed, with the activity randomly selected from four cognitive tasks, including alternate reciting, auditory working memory, clock task, and phonologic fluency. The main outcome variables—TUG duration measured by OPAL accelerometer and cognitive-dual task effect (DTE)—were analyzed using repeated-measures analyses of variance (ANOVA).

**Results:** The number of correct responses when seated were significantly lower in the SOD as compared to the ST (*p* < 0.05) during all cognitive tasks, except the phonologic fluency. During TUG-cognitive, TUG duration in the ST was significantly longer for all cognitive tasks compared with TUG-single (*p* < 0.0001), whereas TUG duration in the SOD was significantly increased only during the phonologic fluency task (*p* < 0.01). In the ST, there was a significant difference in cognitive DTE between the subtraction and the phonologic fluency tasks (*p* < 0.01). The highest cognitive cost was found in the subtraction task, whereas the highest cognitive benefit was shown in the phonologic fluency task. No significant cognitive DTE was found among the cognitive tasks in the SOD.

**Conclusion:** For stroke persons with SOD, phonologic fluency is suitable to be used in the TUG-cognitive assessment. In contrast, subtraction (by 3s) is recommended for the assessment of TUG-cognitive in stroke persons who can perform subtraction.

## Introduction

People who have suffered a stroke exhibit a greater risk of falling than similarly aged individuals (1). Cognition, mobility, and functional performance are major factors that contribute to fall risk and fall-related injury in persons with stroke (2). In addition, the measurement of cognitive function provides essential information that can assist in the prediction of falls (3). Dual-task methodology has been used for assessing cognitive–motor interference (CMI) while walking in various populations, including among persons with stroke. Previous studies reported an association between impaired dual-task performance and the increased risk of falls (4–7). The clinical measures commonly used to assess cognitive-dual task performance during walking include the Stop Walking While Talking Test, the Walking While Talking Test, the Multiple Tasks Test, and the Timed Up and GO cognitive (TUG-cognitive) (8). Among these four clinical tests, the TUG-cognitive is widely used for clinical assessment in stroke patients (9, 10). In the TUG-cognitive, the subjects will be asked to perform TUG [standing up from a chair, walk 3 m, turn, walk back, and sit down (11)] with the addition of cognitive task (subtract by 3s) (12). The TUG cognitive is useful for the evaluation of walking balance. Performing TUG-cognitive has a detrimental effect on functional mobility, of which the additional secondary task increased the time taken to complete the TUG by 22–25% (12). The completion time of TUG-cognitive has 80% sensitivity and 93% specificity for identifying community-dwelling older adults who are prone to falls (12).

A mismatch between available cognitive resources and current task demands often results in the recruitment of additional neural resources, resulting in better or worse task performance (13). Changes in cognitive processes from neural damage following a stroke can be assessed under the dual-task condition. In persons with stroke, the occurrence of CMI while performing simultaneous motor and cognitive tasks results in the impaired performance of one or both tasks (14). Decreased cognitive performance and gait changes can be influenced by the type and the difficulty of the cognitive task (15). Previous studies reported that mental tracking (serial subtraction) was more detrimental to motor performance than discrimination/decision-making and reaction time tasks (16, 17). TUG-cognitive test with serial subtraction was also found to be the most reliable assessment for CMI (18). Decreased motor performance during TUG-serial subtraction, such as taking a longer time during walk, turn, and turn-to-sit tasks, decreased stride length, stride velocity, and increased single leg stance when walking, have been observed in people with stroke (19).

However, some stroke patients are unable to perform subtraction due to damage in the areas of the central nervous system responsible for arithmetic performance (20, 21). It has been reported that quantitative number processing is likely to require bilateral inferior parietal areas, with patients exhibiting damage to this area presenting subtraction deficits. This type of damage limits the use of the TUG-cognitive with serial subtraction test as an assessment for CMI. In patients with subtraction operation difficulties (SOD), it is unclear which cognitive tasks can be used as a substitute for serial subtraction when performing TUG-cognitive assessments. Therefore, the objective of the present study was to determine the type of cognitive task that, when combined with TUG, would lead to the most significant decrease in both motor and cognitive performances in stroke patients not capable of performing subtraction. Data from persons with stroke who did not have a subtraction operation difficulty were also collected as a reference. We hypothesize that adding a cognitive task in the same category as serial subtraction will result in decreased performance in both motor and cognitive assays in stroke patients where the use of subtraction is not possible.

## Materials and Methods

### Participants

Fifty persons with stroke were recruited from two hospitals and five rehabilitation centers based on the following inclusion criteria: diagnosis of cerebrovascular accident, medically stable, and able to walk independently for at least 6 m with or without walking aids. The participants were excluded if they had (1) brainstem or cerebellar lesion, (2) cerebral aneurysm, (3) color blindness, (4) hearing loss, (5) aphasia, (6) severe visual impairment, (7) major depression (as score on 2Q ≥ 1 and score on 9Q questionnaire ≥ 19), (8) orthopedic conditions or pain affecting natural gait, (9) other neurological disorders that sufficiently disturb balance, (10) inadequate language comprehension resulting in an inability to understand instructions, or (11) a comprehension problem (defined as having Mini-Mental State Examination Thai version score of <24 (22). The participants were then classified into two groups based on their ability to perform subtraction by 3s; the groups were designated as able to subtract (ST) and as with SOD. The criteria for inclusion in the SOD group was the inability to perform serial subtraction with one or fewer correct answers out of five within 1 m. Ethical approval was granted by the Institutional Review Board of Srinakharinwirot University. All the participants signed a written informed consent prior to participating in the study.

The sample size calculation for the repeated-measures analyses of variance (ANOVA) was carried out using G^*^power version 3.1. The minimum number of subjects required in each group was 19 persons, based on the estimated values of error probability (α) at 0.05, power (β) at 0.8, six repeated measurements, and the effect size specification of 0.25.

### Measurement Tools

Baseline information including age, gender, diagnosis of stroke, hemiplegic side, time since stroke, occurrence of recurrent stroke, use of walking aids, and number of education years were collected from all participants using a questionnaire. The motor and walking performance of the participants with stroke was determined using the Fug–Meyer Assessment motor subscale of the lower extremity and stride velocity, respectively. The responses to the cognitive tasks were recorded using digital recorders. Two raters evaluated the responses from stroke patients to ensure accurate scoring of the answers and that any repetition was scored once.

For assessing motor performance during TUG-single and TUG-dual, the APDM's Mobility Lab^TM^ (APDM Inc.) was used to collect and store data. A gyroscope (±400°/s range) and an accelerometer (±5 g range) were used to capture the angular movement and the acceleration at a sampling rate of 200 Hz; gait cycles and related events were detected and estimated (23). Four portable 3D inertial sensors were placed on the participant at mid-thoracic, 5th lumbar vertebrae, and left and right ankles. In the TUG protocol, the subjects were instructed to stand up from a chair, walk 3 m with self-selected speed, turn 180°, then walk back, and sit down. During TUG-dual, the participants were asked to perform TUG and cognitive task simultaneously with the instruction to “perform both tasks as well as possible without prioritizing on either gait or cognitive tasks.”

The cognitive tasks can be classified into five categories, including reaction time, mental tracking, working memory, discrimination and decision-making, and verbal fluency (24). Reaction time was not explored in this study as it was impractical in the clinical setting. Based on the results from our pilot study with 29 persons with stroke, the cognitive task from each type that produced the largest detrimental effect on walking (i.e., statistically significant slower gait speed as compared to walking with no cognitive task) was selected. The tasks selected for this study are as follows: (1) subtraction by 3s and alternating reciting (mental tracking category), (2) auditory working memory (working memory category), (3) clock task (discrimination and decision-making category), and (4) phonologic fluency (verbal fluency category) (Table 1).

**Table 1:** Selected cognitive tasks in each category of cognitive dual task.

**Category**	**Task**	**Task selected**	**Instruction**
Mental tracking	-Subtraction by 3 -Spelling backward -Alternate reciting -Arithmetic task	-Subtraction by 3 -Alternate reciting	Reciting out loud serial subtraction of 3, starting from a random three digits number Memorize alphabets heard from recorded voice and recall the alphabets backward from the last to the initial
Working memory	-Easy auditory working memory -Hard auditory working memory -Shopping list task	-Easy auditory working memory	Recalling a series of random numbers
Discrimination and decision making	-Color classify task -Clock task -Stroop test	Clock task	Listening for “time-of-the day” prompt and say aloud “same” or “different” by determining whether the two hands of a clock are on the same or different sides of the clock face
Verbal fluency	-Phonologic fluency (alphabet fluency) -Semantic fluency (category fluency) -Word generation (alphabet and category fluency)	Phonologic fluency	Recalling words with a specific letter given to him/her at the beginning of the test

### Procedures

The participants were asked to performed TUG without a cognitive task (TUG-single) at the beginning of the test. Then, four cognitive tasks were randomly selected until all tasks were performed. The participants in the ST group were asked to perform one additional task of serial subtraction. The participants received standardized verbal instructions regarding the cognitive task procedures and were allowed to practice while sitting on a chair. To avoid learning effects, the contents of the cognitive tasks used during practice were not similar to those performed during the actual analysis (e.g., different numbers, different letters, etc.). After a practice trial, the participants performed the cognitive task when seated (cognitive-single) for 60 s and the number of correct responses was collected by using the tape recorder. Then, they were asked to perform TUG with the same cognitive task category (TUG-dual). Each condition of the task was performed once, and the participants were allowed to rest at the end of each task for 2 min before performing the next task to prevent mental fatigue. To avoid the learning effects, the rater randomly assigned different sets of letters or numbers when assessing the same cognitive category during cognitive-single or TUG-dual. The results from our pilot study ensured that the different sets of letters or numbers selected in this study produced comparable difficulty in the stroke patients. All the test conditions were completed within 1 h.

### Data Analyses

The total TUG duration, stride velocity, and duration of TUG components (sit-to-stand duration, straight walk duration, turn duration, and turn-to-sit duration) were calculated using APDM's Mobility Lab software.

The dual-task effect (DTE) was used to determine the influence of the added cognitive task on the cognitive task performance. To determine the DTE, first, the cognitive correct response rate (CRR), which is the rate of the correct answer from each cognitive task, was calculated by using the following equation (18):

$$\begin{array}{l} \text{CRR}=\frac{\text{number of correct responses}}{\text{Time(s)}} \end{array}$$

Then, the DTE was calculated as (25):

$$\begin{array}{l} \text{DTE}=\frac{\left(\text{dual task }-\text{single task}\right)}{\text{Single task}}\times 100 \end{array}$$

The negative value of the DTE indicates the decrement of cognitive performance under dual-task conditions (which is referred to as “cognitive costs”), while the positive value of the DTE indicates the improvement of cognitive performance under dual-task conditions (which is referred to as “cognitive benefits”) (24).

All statistical analyses were conducted using SPSS statistics software. An independent *t*-test was used for comparing the age, the onset of stroke, the scores for FM-Motor, and the gait speed. The number of education years was compared using the Mann–Whitney *U*-test. Independent *t*-test was also used to compare the number of correct responses at sitting between the ST and the SOD. The level of significance was set at 0.05. Repeated-measures ANOVA was used to examine the effect of cognitive task on TUG durations (total TUG duration and subcomponent TUG durations) and cognitive DTE. In the ST, the design was 1 × 6 (one group and six task conditions), whereas in the SOD the design was 1 × 5 (one group and five task conditions). The level of significance was set at 0.05, with the Bonferroni test used for *post-hoc* analyses.

## Results

**Figure 1:**
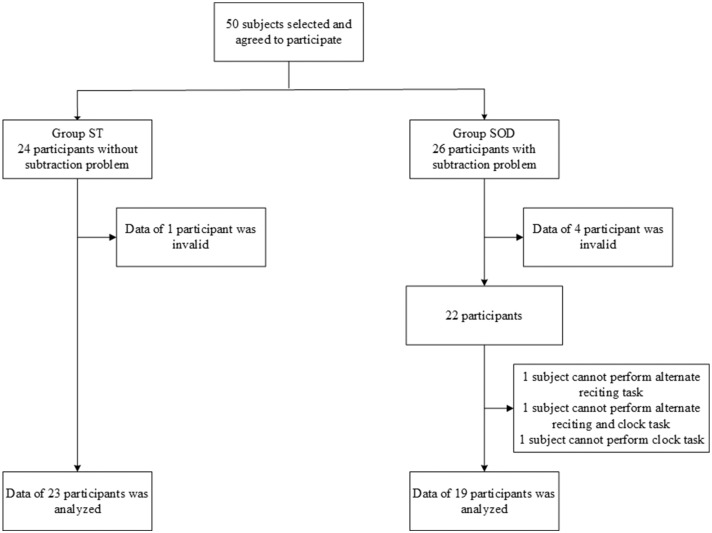
Flow chart showing the number of participants in each group: those who were able to subtract and those with subtraction operation difficulties.

From 50 participants, 24 persons were classified into the ST group and 26 were classified into the SOD group. Five participants were excluded from the analyses because of invalid data, and several other participants were excluded due to their inability to perform all tasks. The final groups consisted of 19 participants in the SOD and 23 participants in the ST (Figure 1). The participants in both groups were similar in age, onset, lower limb function, number of education years, and walking speed (Table 2).

**Table 2:** Demographic characteristic of participants with stroke who were able to subtract (ST) and those with subtraction operation difficulties (SOD).

**Demographic variables**	**ST (*N* = 23)**	**SOD (*N* = 19)**
Age (years)	59.3 ± 10.4	62.0 ± 6.85
Gender (male/female)	13/10	10/9
Hemiparetic side (left/right)	9/14	12/7
Diagnosis of stroke (ischemic/hemorrhagic)	22/1	17/2
Time since stroke (months)	44.13 ± 62.29	42.34 ± 39.69
Recurrent stroke (yes/no)	2/21	3/16
MMSE score	27.52 ± 1.83	25.68 ± 1.8
FM–LE (total = 34)	30.34 ± 4.9	27.74 ± 4.45
Walking speed (m/s)	0.75 ± 0.23	0.70 ± 0.19
Walking aid		
None	22	17
One-point cane	1	1
Three-point cane	–	1
Number of education years (years)	6.87 ± 3.91	5.74 ± 3.11

*Data are presented as mean ± SD; FM–LE, Fugl–Meyer Assessment of Lower Extremity function*.

**Figure 2:**
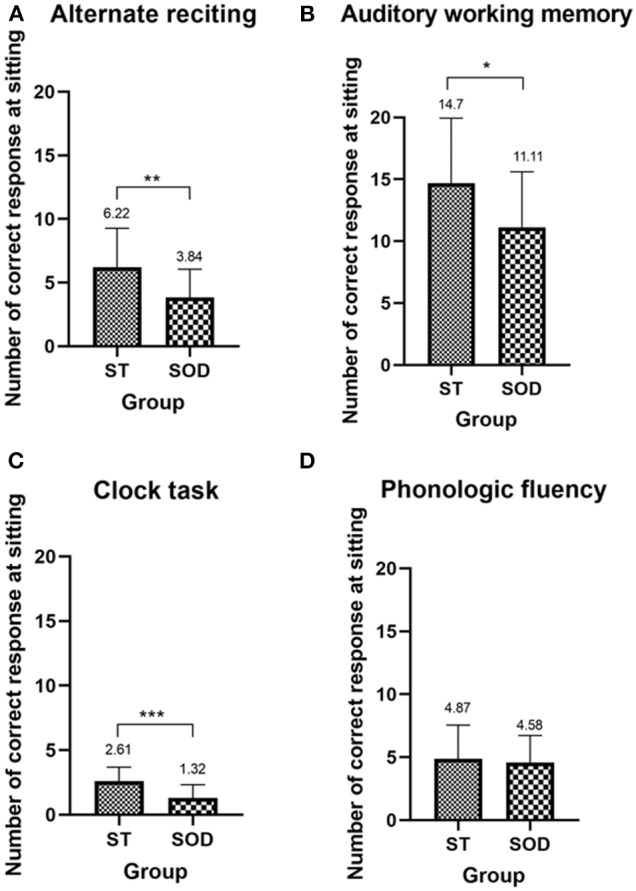
Mean (with SD) of the number of correct responses during sitting of the four cognitive tasks—**(A)** alternate reciting task, **(B)** auditory working memory task, **(C)** clock task, and **(D)** phonologic fluency task—in persons with stroke who were able to subtract and in those with subtraction operation difficulties. *, **, and *** depict a significant difference between the groups at *p* < 0.05, *p* < 0.01, and *p* < 0.001, respectively.

The ability to perform the cognitive-single task in the ST and the SOD was measured from the number of correct responses during sitting (Figure 2). In comparison to the ST, the SOD demonstrated a lower number of correct responses during almost all types of cognitive tasks, including alternate reciting, auditory working memory, and clock task (*p* < 0.01, 0.05, and 0.001, respectively), with the exception of phonologic fluency where no group difference was found.

**Figure 3:**
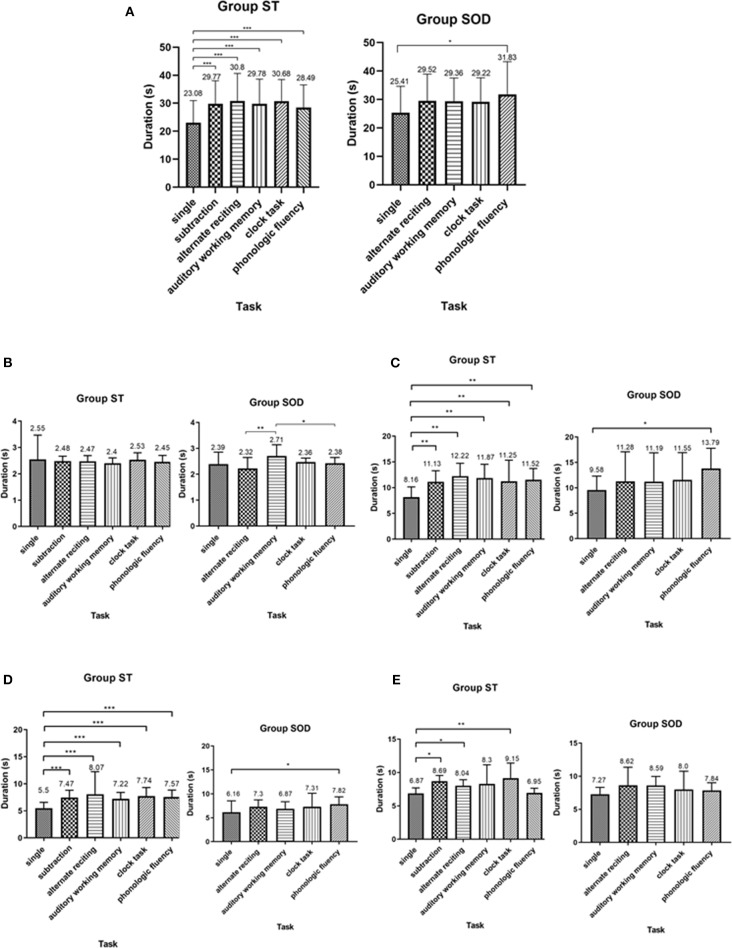
Average duration (with SD) of **(A)** total Timed Up and Go Test duration, **(B)** sit-to-stand duration, **(C)** straight walk duration, **(D)** turning duration, and **(E)** turn-to-sit duration, comparing between cognitive tasks in persons with stroke who were able to subtract and those with subtraction operation difficulties. *depicts a significant difference at *p* < 0.05. **depicts a significant difference at *p* < 0.01. ***depicts a significant difference at *p* < 0.001.

The total TUG duration for both groups during TUG-single and TUG-dual is shown in (Figure 3). Significant effects of adding a cognitive task on total TUG duration during TUG-cognitive [*F*_(5, 110)_ = 23.65, *p* < 0.001 in ST; *F*_(4, 72)_ = 4.47, *p* < 0.01 in SOD] and straight walk duration [*F*_(5, 110)_ = 7.48, *p* < 0.001 in ST; *F*_(4, 72)_ = 3.55, *p* < 0.05 in SOD] were found in both groups. In the ST, total TUG duration and straight walk duration were significantly longer during TUG-dual, for all tasks, as compared to TUG-single (*p* < 0.001). In contrast, total TUG duration and straight walk duration in SOD significantly increased only during the phonologic fluency task (*p* < 0.01 and 0.05, respectively). (Figure 3) also shows the time spent in each of the four components of TUG. There was no significant difference found in duration during the sit-to-stand component between TUG-single and all TUG-dual for both groups. Straight walk duration and turning duration were significantly longer between TUG-single and all TUG-dual in the ST (*p* < 0.01 and 0.0001, respectively), while in the SOD, straight walking duration and turning duration were significantly longer only between TUG-single and TUG-phonologic fluency (*p* < 0.05). Turn-to-sit duration was different from TUG-single during TUG-subtraction (*p* < 0.05), TUG-alternate reciting (*p* < 0.05), and TUG-clock task (*p* < 0.01) in the ST group. In contrast, in the SOD group, no significant difference between tasks was found for turn-to-sit duration.

**Figure 4:**
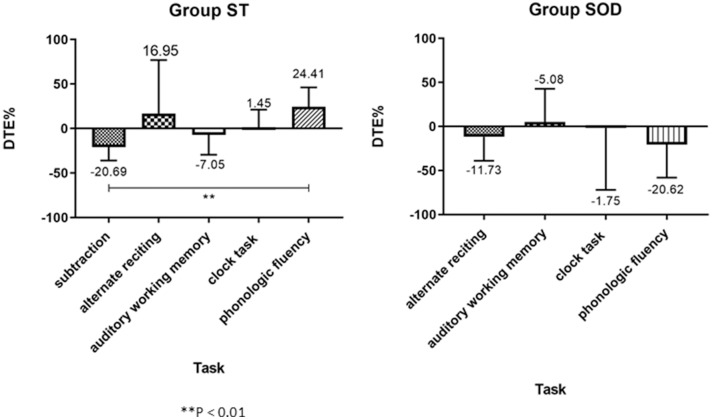
Group average (with SD) of percentage of Cognitive Dual-task Effect (%DTE), comparing between different cognitive tasks in the able to subtract group and in the subtraction operation difficulties group. A positive value means an improvement in cognitive performance (cognitive benefits); a negative value means a decline in cognitive performance (cognitive costs). **depicts a significant difference between tasks at *p* < 0.01.

**Figure 5:**
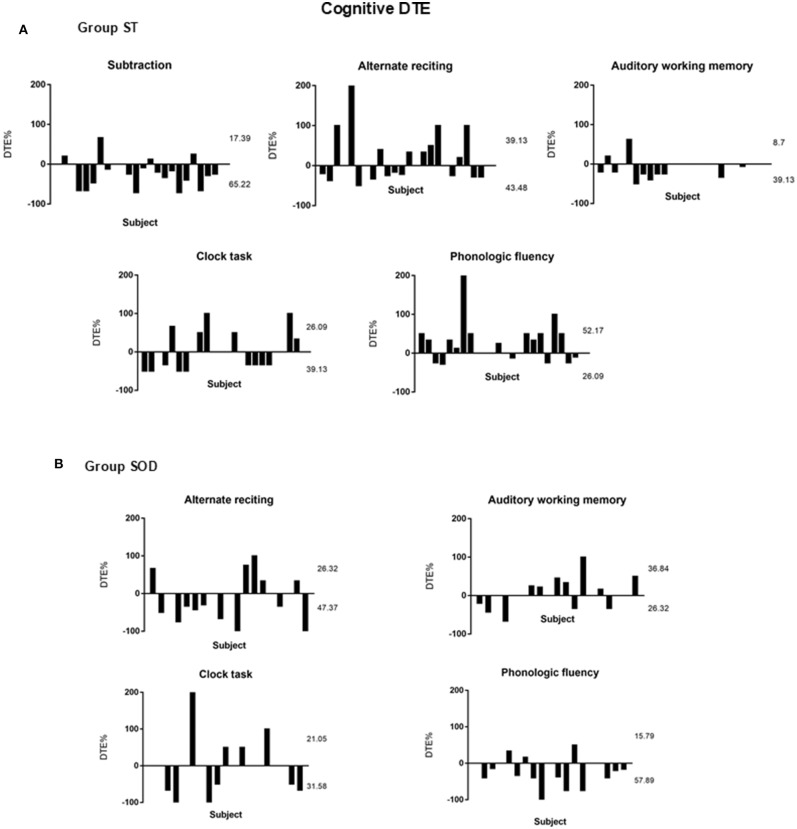
Percentage of Cognitive Dual-task Effect (%DTE) of different cognitive tasks from individual subjects with stroke: **(A)** able to subtract (*N* = 23) and **(B)** subtraction operation difficulties (*N* = 19). A positive value means dual-task benefit, a negative value means dual-task cost, and zero value means no effect. The upper number represents the percentage of participants with cognitive benefit, and the lower number represents the percentage of participants with cognitive cost. The rest of the percentages (not shown in the figure) represented those participants with no cognitive effect (zero).

Three different patterns of the cognitive DTE were found in individuals: decline, no change, and improvement of cognitive performance when compared to cognitive function during sitting (cognitive-single) (Figure 5). The significant effect of adding cognitive task on cognitive performance during TUG-cognitive was found in the ST [*F*_(4, 88)_ = 3.94, *p* < 0.01] but not in the SOD [*F*_(3, 54)_ = 1.15, *p* = 0.34]. The group averages revealed that the highest cognitive costs (as presented by the highest negative value) were from serial subtraction in the ST (−20.69 ± 35.18) and from phonologic fluency task in the SOD (−23.91 ± 41.55) (Figure 4). There were also significant differences in cognitive DTE between subtraction and phonologic fluency tasks in the ST (*p* < 0.01); however, no significant difference was found between these tasks in the SOD (Figure 4). The majority of the participants (65.22%) in the ST group showed a decreased cognitive performance during TUG-dual in the subtraction task compared to that in the cognitive-single. In contrast, many participants in the SOD demonstrated a decline in cognitive performance (57.89%) during the phonologic fluency task (Figure 5).

## Discussion

This study is the first to examine the types of cognitive tasks that cause detrimental effects in both motor and cognitive performances during TUG-dual in persons with stroke and who had a subtraction operational difficulty. Our initial hypothesis that similar types of cognitive tasks would interfere with the cognitive–motor performance in both the ST and the SOD groups did not support this analysis. We found instead that the type of cognitive task played different roles in interfering with the cognitive–motor performance during walking in persons with stroke but capable of performing the subtraction task compared to those who were unable to complete this task.

In persons with stroke but who can perform number subtraction, the subtraction task produced larger negative effects on cognitive–motor performance during cognitive-dual tests than in the other cognitive tasks examined. In a previous study by Patel and Bhatt (16), the subtraction task was also found to cause a higher negative cognitive effect compared to the Stroop task (discrimination and decision-making). This indicates that the type and the complexity of the task are important in dual-task interference (26). We demonstrated in this study that the subtraction task was more complex than phonologic fluency as it resulted in a higher cognitive cost. The difficulty in performing the subtraction task may be due to a requirement for higher neural activity compared to the phonologic fluency task. The subtraction task triggered neural activity in the bilateral inferior parietal network (20, 27), whereas phonologic fluency activated neural networks only in the left inferior frontal cortex and supplementary motor area (28–30). In addition, working memory is required for the subtraction task, and this task is more directly related to executive function than the verbal fluency task (31).

Compared to the subtraction task, the other cognitive tasks used in this study produced more limited detrimental effects on motor and cognitive performances in persons with stroke but who can perform subtraction. The auditory working memory task caused impaired motor and cognitive performance, although at a lower magnitude than the subtraction task. The alternate reciting letter and clock task resulted in decreased TUG performance. However, the effects on cognitive performance were inconclusive as nearly equal numbers of participants were observed with negative and positive effects on cognitive performance. These findings were in agreement with previous studies which reported limited effects from the clock and alternate reciting letter tasks on dual-task gait performance. Dennis et al. (17) reported no change in gait speed in individuals with stroke during the performance of the clock task. Additionally, a report by Liu-Ambrose et al. (32) found that the alternate reciting letter task did not interfere with gait performance in the elderly.

On the contrary, phonologic fluency was found to produce a more detrimental effect than the other cognitive tasks in the group of stroke patients with SOD. We demonstrated in the SOD that total TUG duration was significantly longer only during the phonologic fluency task, with the highest cognitive cost. The differences in cognitive task difficulty may be responsible for this finding in the SOD. The results from the number of correct responses obtained during cognitive-single task (Figure 2) suggested that the ability to perform cognitive tasks in general was lower in the SOD as compared to those who can perform the subtraction task. The phonologic fluency task was considered to be the easiest task among the four testing cognitive tasks for the SOD as they were able to perform this task in a comparable manner as the ST. It was plausible that, when the cognitive tasks were too difficult for the person in the SOD, they may prioritize their attention to the task that they could perform (motor task), leading to no deterioration of the motor task during the three other cognitive tasks: alternate reciting letter, auditory working memory, and clock task. Another explanation lies in the method of calculating the cognitive DTE when the number of correct responses is very low (<3 correct responses) in both the single and the dual tasks. The relative comparison of dual task from single task in this case could lead to a misinterpretation of no cognitive interference during cognitive-dual condition when the actual reason is the inability to perform the cognitive task.

The other possible explanation on why the phonologic fluency led to the highest deterioration of both motor and cognitive performances in the SOD could be because phonologic fluency triggers more neural activities in the supplementary motor area compared to the other cognitive tasks examined. The neural activities in the left intraparietal sulcus, bilateral superior temporal gyrus, and inferior frontal gyrus are activated during the alternate reciting task (33). The working memory task involves an executive attention control mechanism, and this ability is mediated by portions of the prefrontal cortex (34). The activation of the inferior frontal gyrus and the anterior insula bilaterally, the left supramarginal gyrus, and the putamen was noted during the performance of the clock task (35). The supplementary motor area plays an important role in postural control and contributes to the timing and amplitude of the anticipatory postural adjustment of human gait initiation (36). Therefore, the competitive cognitive demand between retrieving specific words within lexical memory and gait control may impair the performance during TUG-dual with the phonologic fluency task.

Some limitations are noted for this study. Due to the heterogeneity of our participants with stroke, the information regarding cognitive DTE during alternate reciting letter, auditory working memory, and clock task was inconclusive. A large sample size is required in the next study to unravel the cognitive DTE during those aforementioned cognitive tasks. Next, the measurement of phonologic fluency is differentially sensitive to age and education (37, 38). The results from this study were obtained from the participants, the majority of whom have a primary education level; thus, the generalization of the results is limited. Lastly, gait pattern, cognitive abilities, and motor and functional outcomes after stroke are correlated with brain lesion site and location (29, 39, 40). A lesion assessment based on CT or MRI images was not taken for all participants. Furthermore, a longitudinal study is required to explore the relationship between performance under TUG-verbal fluency and falls in stroke patients who were not capable of performing subtraction.

Impaired dual-task performance has been associated with an increased risk of falls in people with stroke. The TUG test with serial subtraction is a useful tool to assess cognitive-dual task performance during walking in order to identify persons with stroke who are prone to fall. Apart from the traditional use of arithmetic task such as number subtraction, this study provided the alternative of using phonologic verbal fluency in conjunction with the TUG when assessing the cognitive–motor ability in individuals with stroke and who have SOD. This can be applied in the clinical practice as it will enable the clinicians to customize the cognitive tasks for assessment based on individual limitation so that the fall prevention program can be implemented as early as possible to prevent fall-related consequences in persons with stroke.

## Conclusions

When combined with the TUG, phonologic fluency led to the largest deteriorating effect on dual-task performance in stroke patients with SOD. Therefore, phonologic fluency is suitable to be used in the TUG-cognitive assessment for persons with stroke and who have SOD. In contrast, number subtraction (by 3s) is recommended for the assessment of TUG-cognitive in persons with stroke but who can perform subtraction as it caused the largest reduction in cognitive–motor performance in persons with stroke but who can perform subtraction.

## Data Availability Statement

The datasets generated for this study are available on request to the corresponding author.

## Ethics Statement

The studies involving human participants were reviewed and approved by Institutional Review Board of Srinakharinwirot University. The patients/participants provided their written informed consent to participate in this study.

## Author Contributions

RB conceived and designed the project, funding procurement, and preparation of the final manuscript. AP collected and analyzed the data and wrote the first draft of the manuscript. NC helped with the instrumentation and data analysis. VS helped in the preparation of the final manuscript.

## Conflict of Interest

The authors declare that the research was conducted in the absence of any commercial or financial relationships that could be construed as a potential conflict of interest.
